# Requisites of a Gynæcologist

**Published:** 1886-11

**Authors:** 


### REQUISITES OF A GYNÆCOLOGIST: THE MICHIGAN IDEAL.

Dr. Whitaker is credited with saying "a bald head and bay-window abdomen are important qualities by which to succeed in gyn-

æcology." We have known even this unique combination to fail. The statement is a libel on women. True femininity is in closer sympathy with true virility than it would imply.—*Medical Age,* Detroit.

This, certainly, is a most extraordinary declaration. Now, we would like to ask Brother Mulherron if he speaks from experience, or from mere "hear say?"—if he has found the possession of "true virility" or good "combination" with his gynæcological skill?—and if, in Michigan, both qualities are called into requisition at the same time? If not,—what has the "virility" of the surgeon to do with gynæcology anyhow? Nor do we know by what process of reason—or upon what principle the *Age* concludes that "true virility" is not a concomitant of "a bald head and bay-window-abdomen." A lady patient may see and appreciate the "true" skill of her attendant,—she may admire his "true" politeness, easy, affable manners and other qualities, but, before letting her sympathy go out to him, we are naturally curious to know by what means she can arrive at any estimate of that quality which the *Age* says she so closely sympathizes with?—whether it be "true" or otherwise? The above statement of the *Age* is most ambiguous, and calls for explanation. We think Brother Mulherron's remark is much more of "a libel on women" than that of Dr. Whitaker, and he should apologize. In this matter we are somewhat like Pat's wife, who, when the recruiting officer of the army rejected Pat on the grounds of "physical disability" for a certain delicate ailment, exclaimed: "Begorra! and I thought it was for *foighting* yes wanted him!"

